# Poly[[diaquadi-μ-dicyanamido-nickel(II)] bis­(pyridinium-4-olate)]

**DOI:** 10.1107/S1600536809048880

**Published:** 2009-11-21

**Authors:** Ling-Ling Zheng

**Affiliations:** a College of Chemistry and Chemical Engineering, Chongqing University, Chongqing 400030, People’s Republic of China

## Abstract

The title compound, {[Ni(C_2_N_3_)_2_(H_2_O)_2_]·2C_5_H_5_NO}_*n*_, is a centrosymmetric two-dimensional coordination polymer with a layer (4,4) network structure. The asymmetric unit is compossed of an Ni^II^ atom, which sits on an inversion center, a μ-1,5-bridging dicyanamide anion, a water mol­ecule, and a free 4-hydroxy­pyridine mol­ecule present in the zwitterionic pyridinium-4-olate form. The Ni^II^ atom is coordinated in a slightly distorted N_4_O_2_ octa­hedral geometry by four bridging dicyanamide ligands and two *trans* water mol­ecules. In the crystal, the two-dimensional networks are linked *via* N—H⋯O and O—H⋯O hydrogen bonds, forming a three-dimensional network.

## Related literature

For coordination polymers involving dicyanamide (dca), see: Manson *et al.* (1998 ▶, 2001 ▶); Batten *et al.* (1998 ▶). For nickel(II)–dca complexes, see: Van der Werff *et al.* (2004 ▶); Armentano *et al.* (2006 ▶). For dicyanamide complexes with a co-ligand, see: Batten & Murray (2003 ▶); Manson *et al.* (1998 ▶, 2001 ▶); Miller & Manson (2001 ▶). For dicyanamide complexes with 4-cyano­pyridine as co-ligand, see: Dalai *et al.* (2002 ▶); Du *et al.* (2006 ▶). 
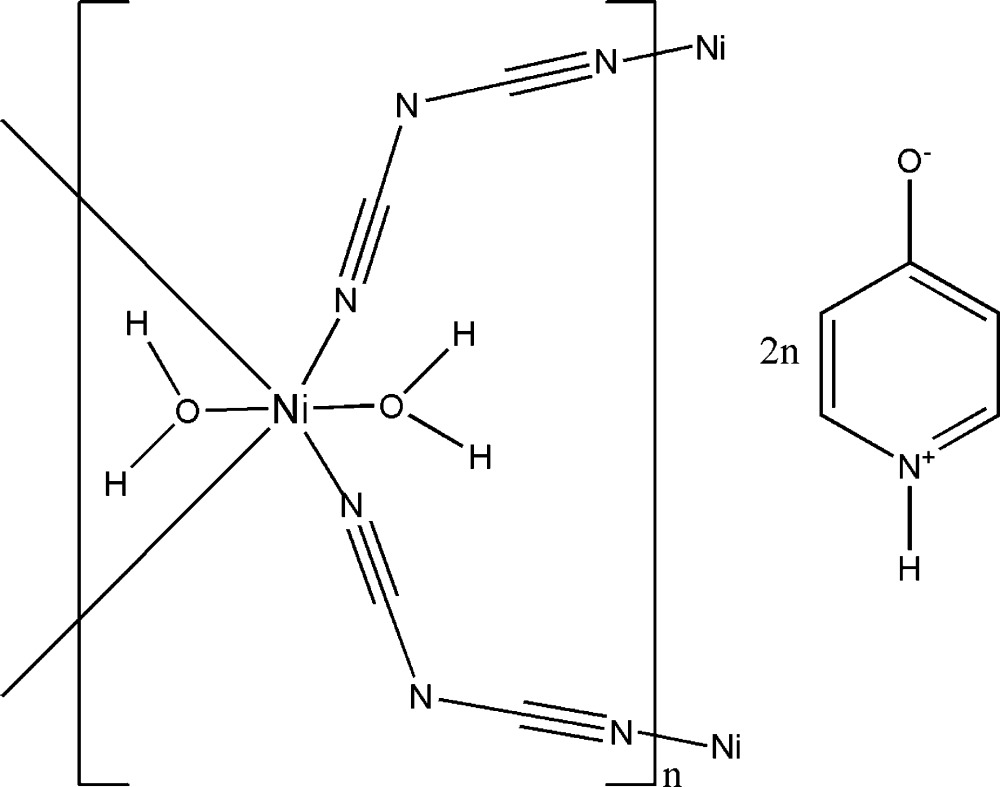


## Experimental

### 

#### Crystal data

[Ni(C_2_N_3_)_2_(H_2_O)_2_]·2C_5_H_5_NO
*M* *_r_* = 417.02
Monoclinic, 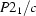

*a* = 7.8598 (6) Å
*b* = 12.8199 (10) Å
*c* = 9.1080 (7) Å
β = 96.7530 (10)°
*V* = 911.37 (12) Å^3^
*Z* = 2
Mo *K*α radiation
μ = 1.10 mm^−1^
*T* = 293 K
0.23 × 0.18 × 0.15 mm

#### Data collection

Bruker SMART CCD area-detector diffractometer
Absorption correction: multi-scan (*SADABS*; Bruker, 2001 ▶) *T*_min_ = 0.788, *T*_max_ = 0.848
6950 measured reflections
1788 independent reflections
1606 reflections with *I* > 2σ(*I*)
*R*_int_ = 0.016

#### Refinement

*R*[*F* ^2^ > 2σ(*F* ^2^)] = 0.028
*wR*(*F* ^2^) = 0.073
*S* = 1.08
1788 reflections
136 parameters
4 restraints
H atoms treated by a mixture of independent and constrained refinement
Δρ_max_ = 0.30 e Å^−3^
Δρ_min_ = −0.16 e Å^−3^

### 

Data collection: *SMART* (Bruker, 2007 ▶); cell refinement: *SAINT* (Bruker, 2007 ▶); data reduction: *SAINT*; program(s) used to solve structure: *SHELXS97* (Sheldrick, 2008 ▶); program(s) used to refine structure: *SHELXL97* (Sheldrick, 2008 ▶); molecular graphics: *XP* in *SHELXTL* (Sheldrick, 2008 ▶); software used to prepare material for publication: *SHELXTL*.

## Supplementary Material

Crystal structure: contains datablocks I, global. DOI: 10.1107/S1600536809048880/su2153sup1.cif

Structure factors: contains datablocks I. DOI: 10.1107/S1600536809048880/su2153Isup2.hkl

Additional supplementary materials:  crystallographic information; 3D view; checkCIF report

## Figures and Tables

**Table 1:** Hydrogen-bond geometry (Å, °)

*D*—H⋯*A*	*D*—H	H⋯*A*	*D*⋯*A*	*D*—H⋯*A*
N4—H*N*4⋯O1^i^	0.858 (16)	2.00 (2)	2.792 (2)	153 (3)
O1*W*—H1*WA*⋯O1^ii^	0.834 (9)	1.912 (11)	2.732 (2)	167 (2)
O1*W*—H1*WB*⋯O1^iii^	0.840 (9)	1.898 (11)	2.715 (2)	164.0 (19)

Symmetry codes: (i) 
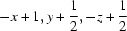
; (ii) 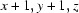; (iii) 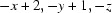.

**Fig. 1.**
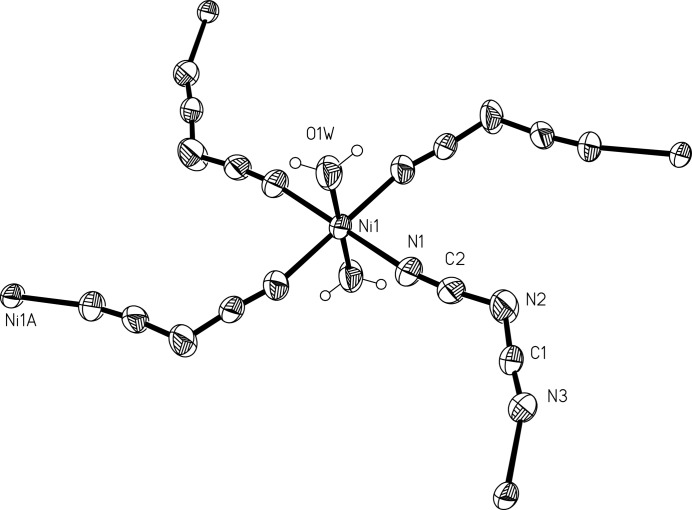
A view of the octahedral coordination geometry of the nickel(II) atom in the title compound. The coligand, present in the 4-pyridium-olate form, has been omitted for clarity. Displacement ellipsoids are shown at the 50% probability level.

**Fig. 2.**
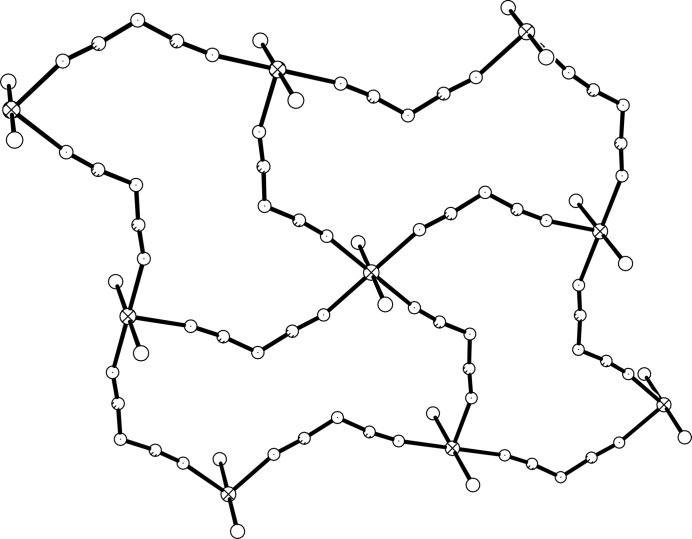
A partial view of the two-dimensional (4,4) network in the title compound.

## supplementary crystallographic information

### Comment

In recent years, coordination polymers involving dicyanamide (dca, N(CN)_2_) have attracted a great deal of attention, not only for their interesting extended architectures but also for their magnetic properties, especially compounds in the *M*(dca)_2_ series (Manson *et al.*, 1998; Batten *et al.*, 1998; Manson *et al.*, 2001). The introduction of coligands has led to dramatic modifications of the crystal structures and magnetic properties (Batten *et al.*, 2003; Miller *et al.*, 2001). When 4-hydroxypyridine was used as a coligand, the title complex was obtained.

The title compound is a centrosymmetric two-dimensional coordination polymer, Fig. 1. The Ni^II^ atom, which is located on an inversion center, is six-coordinated by four N-atoms from four bridging dca ligands and two O-atoms from two water molecules, so exhibiting a distorted octahedral geometry. The Ni—N bond lengths [2.0769 (16) - 2.0785 (15) °] are comparable with those of 2.070 (2) ° found in [(EtPh_3_P)—Ni(dca)_3_] (Van der Werff *et al.*, 2004), and 2.0715 (12) ° found in [Ni(dca)_2_(phen)] (Armentano *et al.*, 2006). The coligand used, 4-hydroxypyridine, is present in the pyridinium-4-olate form and is not coordinted to the metal atom.

In the crystal structure, the µ-1,5 dca ligand links neighboring Ni^II^ atoms, forming a two-dimensional (4,4) network (Fig. 2). This resembles the situation in [Mn(dca)_2_(4-cyanopyridine)_2_]_n_ (Dalai *et al.*, 2002) and [Co_2_(dca)_4_(4-cyanopyridine)_4_]_n_(Du *et al.*, 2006), but the structures are not isomorphous and here the co-ligands are coordinated to the metal atoms. However, as in the title compound, the layer-like structures are linked through hydrogen bonding interactions. In the title compound this involves the water molecules and the pyridinium-4-olate groups (Table 1).

### Experimental

To a methanol solution (20 mL) of nickel nitrate (0.145 g, 0.5 mmol) and 4-hydroxypyridine (0.095 g, 1.0 mmol), a water solution (5 ml) of dca (0.089 g, 1.0 mmol) was added slowly with stirring over 30 min at rt. The clear solution obtained was filtered and the filtrate left to evaporated at rt. After a few days, green single crystals were obtained (yield: 5%).

### Refinement

The NH and water H-atoms were located in difference electron-density maps and freely refined: N-H = 0.858 (16) Å, O-H = 0.834 (9) & 0.840 (9) Å. The C-bound H-atoms were included in calculated positions and treated as riding: C-H = 0.93 Å, with *U*_iso_(H) = 1.2*U*_eq_(C).

### Crystal data

**Table:**

[Ni(C_2_N_3_)_2_(H_2_O)_2_]·2C_5_H_5_NO	*F*(000) = 428
*M**_r_* = 417.02	*D*_x_ = 1.520 Mg m^−^^3^
Monoclinic, *P*2_1_/*c*	Mo *K*α radiation, λ = 0.71073 Å
Hall symbol: -P 2ybc	Cell parameters from 1788 reflections
*a* = 7.8598 (6) Å	θ = 2.6–26.0°
*b* = 12.8199 (10) Å	µ = 1.10 mm^−^^1^
*c* = 9.1080 (7) Å	*T* = 293 K
β = 96.753 (1)°	Block, green
*V* = 911.37 (12) Å^3^	0.23 × 0.18 × 0.15 mm
*Z* = 2	

### Data collection

**Table:**

Bruker SMART CCD area-detector diffractometer	1788 independent reflections
Radiation source: fine-focus sealed tube	1606 reflections with *I* > 2σ(*I*)
graphite	*R*_int_ = 0.016
φ and ω scans	θ_max_ = 26.0°, θ_min_ = 2.6°
Absorption correction: multi-scan (*SADABS*; Bruker, 2001)	*h* = −8→9
*T*_min_ = 0.788, *T*_max_ = 0.848	*k* = −15→15
6950 measured reflections	*l* = −10→11

### Refinement

**Table:**

Refinement on *F*^2^	Primary atom site location: structure-invariant direct methods
Least-squares matrix: full	Secondary atom site location: difference Fourier map
*R*[*F*^2^ > 2σ(*F*^2^)] = 0.028	Hydrogen site location: inferred from neighbouring sites
*wR*(*F*^2^) = 0.073	H atoms treated by a mixture of independent and constrained refinement
*S* = 1.08	*w* = 1/[σ^2^(*F*_o_^2^) + (0.0362*P*)^2^ + 0.2827*P*] where *P* = (*F*_o_^2^ + 2*F*_c_^2^)/3
1788 reflections	(Δ/σ)_max_ < 0.001
136 parameters	Δρ_max_ = 0.30 e Å^−^^3^
4 restraints	Δρ_min_ = −0.16 e Å^−^^3^

### Special details

**Table:**

Geometry. All e.s.d.'s (except the e.s.d. in the dihedral angle between two l.s. planes) are estimated using the full covariance matrix. The cell e.s.d.'s are taken into account individually in the estimation of e.s.d.'s in distances, angles and torsion angles; correlations between e.s.d.'s in cell parameters are only used when they are defined by crystal symmetry. An approximate (isotropic) treatment of cell e.s.d.'s is used for estimating e.s.d.'s involving l.s. planes.
Refinement. Refinement of *F*^2^ against ALL reflections. The weighted *R*-factor *wR* and goodness of fit *S* are based on *F*^2^, conventional *R*-factors *R* are based on *F*, with *F* set to zero for negative *F*^2^. The threshold expression of *F*^2^ > σ(*F*^2^) is used only for calculating *R*-factors(gt) *etc*. and is not relevant to the choice of reflections for refinement. *R*-factors based on *F*^2^ are statistically about twice as large as those based on *F*, and *R*- factors based on ALL data will be even larger.

### Fractional atomic coordinates and isotropic or equivalent isotropic displacement parameters (Å^2^)

**Table:**

	*x*	*y*	*z*	*U*_iso_*/*U*_eq_	
Ni1	1.0000	1.0000	0.0000	0.03617 (12)	
C1	0.9985 (2)	0.59861 (12)	0.1821 (2)	0.0424 (4)	
C2	0.9988 (3)	0.75355 (14)	0.0636 (2)	0.0500 (5)	
C3	0.6251 (3)	0.38083 (17)	0.1568 (3)	0.0771 (7)	
H3	0.7156	0.4248	0.1417	0.092*	
C4	0.6426 (3)	0.27675 (16)	0.1384 (3)	0.0657 (6)	
H4	0.7434	0.2503	0.1088	0.079*	
C5	0.5095 (2)	0.20841 (13)	0.1638 (2)	0.0458 (4)	
C6	0.3615 (3)	0.25592 (16)	0.2048 (3)	0.0678 (7)	
H6	0.2686	0.2145	0.2216	0.081*	
C7	0.3507 (3)	0.36025 (17)	0.2205 (3)	0.0683 (6)	
H7	0.2513	0.3896	0.2486	0.082*	
N1	0.9923 (2)	0.84211 (11)	0.05067 (19)	0.0499 (4)	
N2	1.0097 (3)	0.65203 (13)	0.0627 (2)	0.0801 (7)	
N3	0.9881 (2)	0.54506 (12)	0.28000 (17)	0.0481 (4)	
N4	0.4816 (3)	0.42170 (13)	0.1960 (2)	0.0641 (5)	
O1	0.52314 (18)	0.10983 (10)	0.15018 (18)	0.0600 (4)	
O1W	1.26183 (19)	0.99136 (10)	0.01412 (19)	0.0535 (4)	
HN4	0.473 (4)	0.4871 (14)	0.214 (4)	0.091 (10)*	
H1WA	1.330 (2)	1.0346 (15)	0.057 (2)	0.070 (7)*	
H1WB	1.313 (2)	0.9513 (14)	−0.039 (2)	0.061 (6)*	

### Atomic displacement parameters (Å^2^)

**Table:**

	*U*^11^	*U*^22^	*U*^33^	*U*^12^	*U*^13^	*U*^23^
Ni1	0.0457 (2)	0.02211 (17)	0.0425 (2)	−0.00065 (11)	0.01317 (14)	−0.00113 (11)
C1	0.0567 (11)	0.0236 (7)	0.0491 (10)	0.0012 (7)	0.0155 (8)	−0.0022 (7)
C2	0.0751 (14)	0.0337 (10)	0.0449 (10)	−0.0002 (9)	0.0223 (9)	0.0043 (8)
C3	0.0755 (16)	0.0437 (12)	0.117 (2)	−0.0220 (11)	0.0303 (15)	−0.0086 (12)
C4	0.0528 (12)	0.0457 (11)	0.1031 (18)	−0.0087 (9)	0.0282 (12)	−0.0134 (11)
C5	0.0489 (10)	0.0324 (8)	0.0575 (11)	−0.0035 (7)	0.0118 (8)	−0.0082 (8)
C6	0.0577 (13)	0.0430 (11)	0.1088 (19)	−0.0096 (9)	0.0355 (13)	−0.0184 (11)
C7	0.0627 (14)	0.0477 (11)	0.0971 (18)	0.0079 (10)	0.0204 (13)	−0.0200 (11)
N1	0.0674 (11)	0.0284 (8)	0.0565 (9)	−0.0011 (7)	0.0182 (8)	0.0034 (7)
N2	0.163 (2)	0.0272 (8)	0.0577 (11)	0.0077 (10)	0.0439 (12)	0.0057 (8)
N3	0.0675 (11)	0.0315 (8)	0.0472 (9)	0.0000 (7)	0.0144 (7)	0.0043 (7)
N4	0.0811 (14)	0.0302 (9)	0.0808 (13)	0.0004 (8)	0.0082 (11)	−0.0084 (8)
O1	0.0604 (9)	0.0298 (6)	0.0932 (11)	−0.0021 (6)	0.0234 (8)	−0.0126 (7)
O1W	0.0447 (7)	0.0448 (8)	0.0725 (10)	−0.0022 (6)	0.0135 (7)	−0.0208 (7)

### Geometric parameters (Å, °)

**Table:**

Ni1—O1W^i^	2.0498 (15)	C4—C5	1.405 (3)
Ni1—O1W	2.0498 (15)	C4—H4	0.9300
Ni1—N3^ii^	2.0769 (16)	C5—O1	1.276 (2)
Ni1—N3^iii^	2.0769 (16)	C5—C6	1.402 (3)
Ni1—N1	2.0785 (15)	C6—C7	1.349 (3)
Ni1—N1^i^	2.0785 (15)	C6—H6	0.9300
C1—N3	1.136 (2)	C7—N4	1.335 (3)
C1—N2	1.297 (3)	C7—H7	0.9300
C2—N1	1.142 (2)	N3—Ni1^iv^	2.0769 (15)
C2—N2	1.304 (2)	N4—HN4	0.858 (16)
C3—N4	1.330 (3)	O1W—H1WA	0.834 (9)
C3—C4	1.354 (3)	O1W—H1WB	0.840 (9)
C3—H3	0.9300		
			
O1W^i^—Ni1—O1W	180.0	C3—C4—H4	119.8
O1W^i^—Ni1—N3^ii^	91.34 (7)	C5—C4—H4	119.8
O1W—Ni1—N3^ii^	88.66 (7)	O1—C5—C6	122.59 (18)
O1W^i^—Ni1—N3^iii^	88.66 (7)	O1—C5—C4	121.93 (18)
O1W—Ni1—N3^iii^	91.34 (7)	C6—C5—C4	115.49 (17)
N3^ii^—Ni1—N3^iii^	180.00 (2)	C7—C6—C5	121.6 (2)
O1W^i^—Ni1—N1	90.65 (6)	C7—C6—H6	119.2
O1W—Ni1—N1	89.35 (6)	C5—C6—H6	119.2
N3^ii^—Ni1—N1	86.80 (6)	N4—C7—C6	120.5 (2)
N3^iii^—Ni1—N1	93.20 (6)	N4—C7—H7	119.7
O1W^i^—Ni1—N1^i^	89.35 (6)	C6—C7—H7	119.7
O1W—Ni1—N1^i^	90.65 (6)	C2—N1—Ni1	171.49 (17)
N3^ii^—Ni1—N1^i^	93.20 (6)	C1—N2—C2	120.69 (18)
N3^iii^—Ni1—N1^i^	86.80 (6)	C1—N3—Ni1^iv^	157.84 (15)
N1—Ni1—N1^i^	180.0	C3—N4—C7	120.46 (18)
N3—C1—N2	174.66 (19)	C3—N4—HN4	121 (2)
N1—C2—N2	173.5 (2)	C7—N4—HN4	118 (2)
N4—C3—C4	121.5 (2)	Ni1—O1W—H1WA	125.4 (15)
N4—C3—H3	119.2	Ni1—O1W—H1WB	122.9 (14)
C4—C3—H3	119.2	H1WA—O1W—H1WB	110.3 (15)
C3—C4—C5	120.4 (2)		

Symmetry codes: (i) −*x*+2, −*y*+2, −*z*; (ii) *x*, −*y*+3/2, *z*−1/2; (iii) −*x*+2, *y*+1/2, −*z*+1/2; (iv) −*x*+2, *y*−1/2, −*z*+1/2.

### Hydrogen-bond geometry (Å, °)

**Table:**

*D*—H···*A*	*D*—H	H···*A*	*D*···*A*	*D*—H···*A*
N4—HN4···O1^v^	0.86 (2)	2.00 (2)	2.792 (2)	153 (3)
O1W—H1WA···O1^vi^	0.83 (1)	1.91 (1)	2.732 (2)	167 (2)
O1W—H1WB···O1^vii^	0.84 (1)	1.90 (1)	2.715 (2)	164 (2)

Symmetry codes: (v) −*x*+1, *y*+1/2, −*z*+1/2; (vi) *x*+1, *y*+1, *z*; (vii) −*x*+2, −*y*+1, −*z*.
